# Complete Genome Sequence of Chlamydia abortus MRI-10/19, Isolated from a Sheep Vaccinated with the Commercial Live *C. abortus* 1B Vaccine Strain

**DOI:** 10.1128/MRA.00203-21

**Published:** 2021-05-06

**Authors:** Morag Livingstone, Sergio Gastón Caspe, David Longbottom

**Affiliations:** a Moredun Research Institute, Edinburgh, Midlothian, United Kingdom; b Instituto Nacional de Tecnología Agropecuaria, Mercedes, Corrientes, Argentina; University of Maryland School of Medicine

## Abstract

We report the complete genome sequence of Chlamydia abortus MRI-10/19, recovered from the infected placenta of a sheep that had been vaccinated with the commercial live attenuated *C. abortus* 1B vaccine strain. Comparative analysis revealed 1 single nucleotide polymorphism (SNP) difference and 4 indels compared to the vaccine strain.

## ANNOUNCEMENT

Chlamydia abortus, an obligate intracellular Gram-negative bacterium and a cause of enzootic abortion of ewes (EAE), is responsible for late-term abortion, stillbirths, and the birth of weak offspring (1). In Europe, the disease is controlled using the commercial live *C. abortus* 1B vaccine (2), which has been associated with infections and cases of abortion in vaccinated ewes (3–7).

Here, we report the complete genome sequence of *C. abortus* strain MRI-10/19, isolated from the placenta of a sheep that had been vaccinated with the commercial 1B strain (Cevac Chlamydia, Ceva Animal Health Ltd.) and showed evidence of gross lesions typical of EAE (8). The strain was isolated from pooled placental tissue following the inoculation of ground-up and filtered material onto HEp-2 cells (8). Elementary bodies were purified from infected cultures (9) and genomic DNA extracted using a DNeasy blood and tissue kit (Qiagen). The DNA concentration and purity were determined using a Qubit double-stranded DNA (dsDNA) broad-range (BR) assay kit (Invitrogen) and a NanoDrop One spectrophotometer (Thermo Scientific), respectively.

A genomic DNA library was prepared using the Nextera XT library preparation kit for sequencing on an Illumina HiSeq platform using a 250-bp paired-end protocol. The reads were adapter trimmed using Trimmomatic v0.30 (10) with a sliding window quality cutoff of Q15. Taxonomic classification to the species level as *C. abortus* was confirmed using Kraken v2.1.1 (11). A long-read genomic DNA library was prepared using a rapid barcoding kit (SQK-RBK004) and sequenced in a MinION FLO-MIN106 flow cell (MinKNOW v20.10.3), with integrated live base calling provided by Guppy v4.4.1 (Oxford Nanopore Technologies). The reads were filtered using Filtlong v0.2.0 (12) with a minimum cutoff of 5,000 bp. All trimmed raw data analysis was performed on the Galaxy platform (http://usegalaxy.org.au/) (13). The read quality was checked using FastQC (Galaxy v0.72+galaxy1) (14) and NanoPlot (Galaxy v1.28.2+galaxy1) (15). Sequencing resulted in 232,402 Illumina paired-end reads (average read length, 242 bp) and 363,047 filtered Nanopore reads (average read length, 6,842 bp; read *N*_50_, 6,663 bp). Hybrid *de novo* assembly of the Illumina and Nanopore raw reads was carried out using the Unicycler pipeline (Galaxy v.0.4.8.0) (16), producing a single contig comprising a circular chromosome of 1,144,464 bp with 39.9% GC content and oriented at the *hemB* gene. The average genome coverages for the Illumina and Nanopore read sequences were calculated as 97.4× and 2,107.7×, respectively, using BWA-MEM (Galaxy v0.7.17.1) (17). The assembly metrics were calculated using QUAST (Galaxy v5.0.2+galaxy1) (18). Annotation using the NCBI Prokaryotic Genome Annotation Pipeline v5.0 (19) identified 1,004 predicted genes and 1 rRNA operon. Default settings were used throughout for all utilized software packages.

Pairwise comparative analysis of the assembled sequence with the *C. abortus* 1B Cevac vaccine strain (GenBank accession number LN589721.1) using Mauve v2015.02.26 (20) identified one SNP at position 131000 and indels in homopolymeric (poly-G) tracts at positions 320135, 684576, 687229, and 991350, identifying the strain as originating from infection with the commercial live vaccine and being responsible for the reported pathological placental lesions (8).

### Data availability.

The *C. abortus* MRI-10/19 genome sequence is available in GenBank/EMBL/DDBJ under accession number CP070224. The raw sequence reads are available under BioProject accession number PRJNA700999.
